# The first survey addressing patients with BMI over 50: a survey of 789 bariatric surgeons

**DOI:** 10.1007/s00464-021-08979-w

**Published:** 2022-01-21

**Authors:** Mohammad Kermansaravi, Panagiotis Lainas, Shahab Shahabi Shahmiri, Wah Yang, Amirhossein Davarpanah Jazi, Ramon Vilallonga, Luciano Antozzi, Chetan Parmar, Radwan Kassir, Sonja Chiappetta, Lorea Zubiaga, Antonio Vitiello, Kamal Mahawar, Miguel Carbajo, Mario Musella, Scott Shikora

**Affiliations:** 1 Minimally Invasive Surgery Research Center, Division of Minimally Invasive and Bariatric Surgery, Department of Surgery, Rasool-e Akram Hospital, Iran University of Medical Sciences, Tehran, Iran; 2 Center of Excellence of European Branch of International Federation for Surgery of Obesity, Hazrat_e Rasool Hospital, Tehran, Iran; 3 Department of Minimally Invasive Digestive Surgery, Antoine-Béclère Hospital, Paris-Saclay University, Clamart, France; 4 Metropolitan Hospital of Athens, HEAL Academy, Athens, Greece; 5 Minimally Invasive Surgery Research Center, Iran University of Medical Sciences, Tehran, Iran; 6 Department of Metabolic and Bariatric Surgery, The First Affiliated Hospital of Jinan University, Guangzhou, China; 7 Department of General Surgery, Shariati Hospital, Isfahan, Iran; 8 Endocrine, Metabolic and Bariatric Unit, Department of General and Digestive Surgery, Vall d’Hebron University Hospital, Universitat Autònoma de Barcelona, Center of Excellence for the EAC-BC, Passeig de la Vall d’Hebron 119-129, 08035 Barcelona, Spain; 9 ELSAN, Clinique Saint Michel, Centre Chirurgical de L’Obésité, Toulon, France; 10 Centro de Cirugías Especiales, Bahía Blanca, Argentina; 11 Department of Surgery, The Whittington Health NHS Trust, London, UK; 12 University College London Medical School, London, UK; 13 Department of Digestive Surgery, Centre Hospitalier Universitaire Félix Guyon, St Denis de la Réunion, France; 14 Obesity and Metabolic Surgery Unit, Ospedale Evangelico Betania, Naples, Italy; 15 Miguel Hernandez of Elche University, Alicante, Spain; 16 Advanced Biomedical Sciences Department, Naples “Federico II” University, AOU “Federico II”, Via S. Pansini 5, 80131 Naples, Italy; 17 South Tyneside and Sunderland Foundation NHS Trust, Sunderland, UK; 18 Centre of Excellence for the Study and Treatment of Obesity and Diabetes, Valladolid, Spain; 19 Brigham and Women’s Hospital, Harvard Medical School, Boston, MA USA

**Keywords:** Bariatric surgery, Super obesity, BMI over 50, Survey

## Abstract

**Background:**

Bariatric surgery in patients with BMI over 50 kg/m^2^ is a challenging task. The aim of this study was to address main issues regarding perioperative management of these patients by using a worldwide survey.

**Methods:**

An online 48-item questionnaire-based survey on perioperative management of patients with a BMI superior to 50 kg/m^2^ was ideated by 15 bariatric surgeons from 9 different countries. The questionnaire was emailed to all members of the International Federation of Surgery for Obesity (IFSO). Responses were collected and analyzed by the authors.

**Results:**

789 bariatric surgeons from 73 countries participated in the survey. Most surgeons (89.9%) believed that metabolic/bariatric surgery (MBS) on patients with BMI over 50 kg/m^2^ should only be performed by expert bariatric surgeons. Half of the participants (55.3%) believed that weight loss must be encouraged before surgery and 42.6% of surgeons recommended an excess weight loss of at least 10%. However, only 3.6% of surgeons recommended the insertion of an Intragastric Balloon as bridge therapy before surgery. Sleeve Gastrectomy (SG) was considered the best choice for patients younger than 18 or older than 65 years old. SG and One Anastomosis Gastric Bypass were the most common procedures for individuals between 18 and 65 years. Half of the surgeons believed that a 2-stage approach should be offered to patients with BMI > 50 kg/m^2^, with SG being the first step. Postoperative thromboprophylaxis was recommended for 2 and 4 weeks by 37.8% and 37.7% of participants, respectively.

**Conclusion:**

This survey demonstrated worldwide variations in bariatric surgery practice regarding patients with a BMI superior to 50 kg/m^2^. Careful analysis of these results is useful for identifying several areas for future research and consensus building.

**Supplementary Information:**

The online version contains supplementary material available at 10.1007/s00464-021-08979-w.

According to the World Health Organization, in 2016, 650 million of adults were obese [1]. Despite all efforts to support prevention campaigns and nonsurgical treatments (e.g., lifestyle modifications, medications, behavioral therapy and diets) [2, 3], the prevalence of obesity is steadily increasing worldwide becoming an important health and financial burden. Bariatric surgery represents a safe and effective treatment for patients with morbid obesity and its related diseases [4–6]. Over the past few decades, diffusion and standardization of principal bariatric procedures has led to a significant reduction of peri- and postoperative complications [7–10]. However, these interventions may be challenging in patients with BMI > 50 kg/m^2^ with higher risks [11–15]. Indeed, the large amount of visceral fat makes the surgical approach more technically demanding, while, on the other hand, the advanced stage of obesity often carries severe comorbidities that could worsen perioperative outcomes. Moreover, subjects with BMI over 50 kg/m^2^ are poor respondents in terms of weight loss and metabolic outcomes when compared to patients with lower values of BMI. Despite all these concerns, there is no consensus on the perioperative management of these patients.

The aim of this study was to address the issue of perioperative management of patients with BMI over 50 kg/m^2^ undergoing bariatric surgery using a specific well-designed survey.

To comply with new policy of the International Federation for the Surgery for Obesity and Related Metabolic Disorders (IFSO), the term “super-obese” was not used.

## Methods

A confidential, voluntary, online questionnaire-based survey was sent to the bariatric community through IFSO mailing list and social media. The 48 questions were conceived (Online Appendix 1) by 15 bariatric surgeons from 9 different countries in an attempt to address existing controversies and challenges on perioperative management of patients with BMI over 50 kg/m^2^. The questionnaire was divided into 4 parts: (1) preoperative general evaluation and management, (2) preoperative weight loss management, (3) surgical technique details, and (4) postoperative management (see Online Appendix 1).

Each part included multiple-choice questions with a comment box at the end. The survey started on January 15th and closed for analysis on March 2nd, 2021. The survey was emailed twice to IFSO members and the link was also freely shared on social and scientific media (Facebook™, Researchgate™, Whatsapp™, LinkedIn™ and WeChat®) and through personal networks and societies of bariatric and metabolic surgeons of a few countries. Responses were collected and reported as percentages, while graphs were used for representation where applicable. Being a survey among surgeons, Institution Review Board approval was not needed for this study.

Surgical procedures included in this survey were: (i) sleeve gastrectomy (SG): removal of about 80% of the stomach leaving a tube-shaped stomach; (ii) one anastomosis gastric bypass (OAGB): division of the upper part of the stomach into a tube, similar to the top three quarters of a sleeve, and then joined to a loop of intestine; (iii) Roux-en-Y gastric bypass (RYGB): creation of a small pouch of the upper stomach by stapling and direct attachment to it to part of the small intestine called the Roux limb ("Y" shape); (iv) laparoscopic adjustable gastric banding (LAGB): placement of an adjustable band around the top part of the stomach; (v) single anastomosis duodeno-ileal bypass with sleeve gastrectomy (SADI-S): creation of a sleeve gastrectomy followed by an end-to-side duodeno-ileal diversion; (vi) biliopancreatic diversion with duodenal switch (BPD/DS): creation of a sleeve gastrectomy and connection of the end portion of the intestine to the duodenum near the stomach.

## Results

A total of 820 participants responded to the survey. However, 31 were not bariatric surgeons and their responses were excluded. Therefore, a final number of 789 responses were analyzed.

Three hundred and twenty surgeons (40.6%) had performed more than 1000 metabolic and bariatric surgeries (MBS), while 138 (17.5%), 219 (27.8%), and 112 (14.2%) had done 501 to 1000, 100 to 500, and Less than 100 MBS, respectively.

### Nationality of participants

Bariatric surgeons from 73 countries took part in the survey. Online Table 1 shows the number and percentages of participants from each nation in alphabetical order.

### Preoperative evaluations for patients with BMIs over 50 kg/m^2^

A total of 709 (89.9%) surgeons believe that MBS on patients with BMI over 50 kg/m^2^, should only be performed by expert bariatric surgeons and 621 (78.7%) of them also stated that only experienced bariatric anesthesiologists should be involved. Most surgeons (79.2%) do not use any surgical risk scoring system for these patients. Almost 73% of participants had performed MBS on adolescent patients (under 18 years old) with BMI over 50 kg/m^2^, while72.2% of them reported to have carried out bariatric interventions on elderly patients (over 65 years old). Preoperative eating and psychological disorders are routinely assessed by 73.7% of surgeons. About 76.5% of participants recommend preoperative and postoperative CPAP only in selected cases with sleep apnea. Responses on preoperative evaluation are summarized in Online Appendix 2.

### Preoperative weight loss management in patients with BMIs over 50 kg/m^2^

Weight loss must be encouraged before surgery for 55.34% of participants and 42.6% recommend an excess weight loss of at least 10%. Only 3.6% of surgeons recommended insertion of an Intragastric Balloon (IGB) for patients and most of them (62.2%) only in selected cases. Likewise, 66.6% recommended preoperative Liraglutide only for selected cases. Forty-two percent of participants ask patients to start a very low-calorie diet (VLCD) 4 weeks prior to surgery for liver shrinkage and 23.3% of them only for patients with hepatomegaly. On the other hand, 38.3% believed rate of weight loss is not important to proceed with surgery. Data on preoperative weight loss management are summarized in Online Appendix 3.

### Choice of surgical procedure

**Fig. 1:**
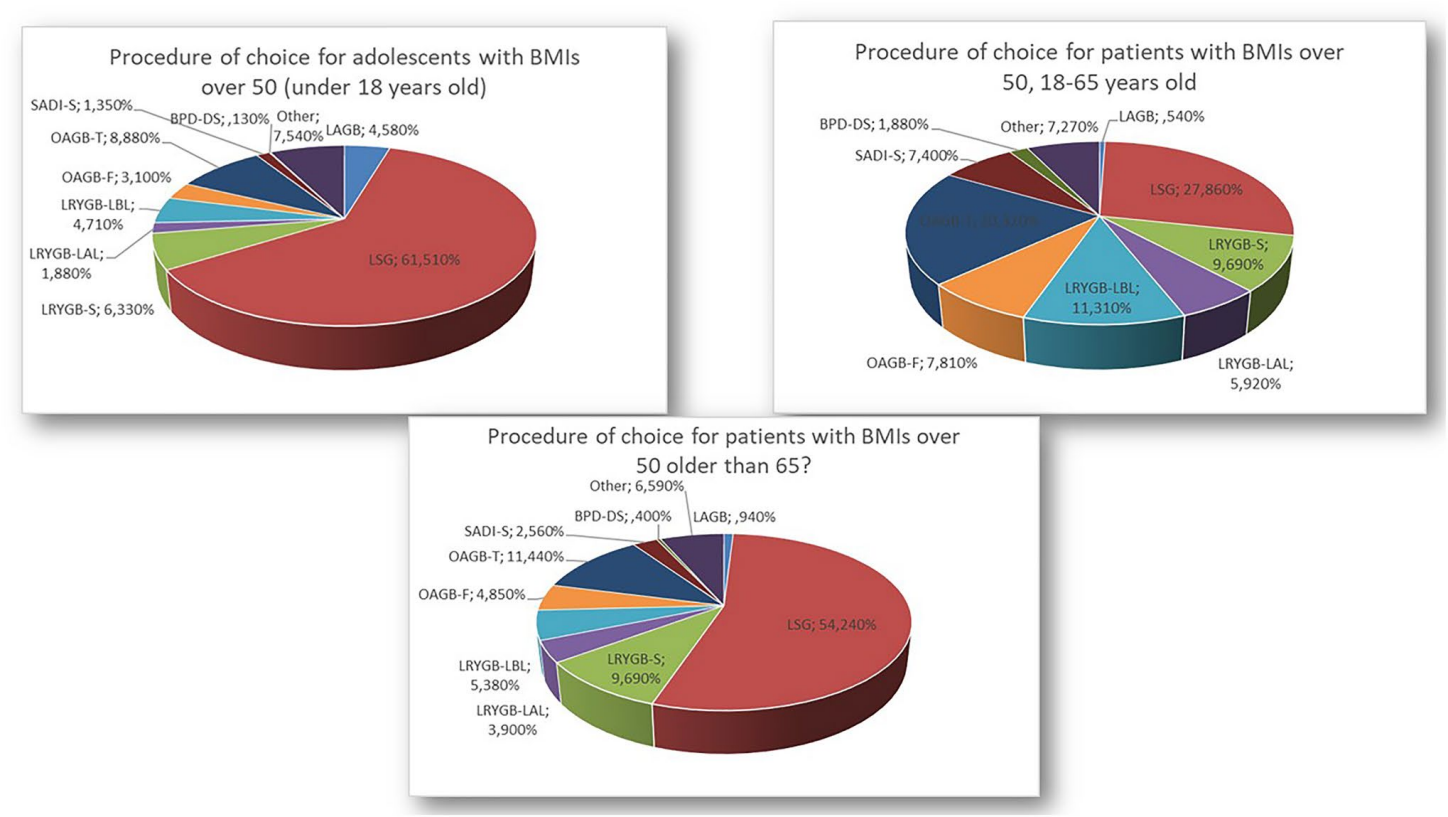
Procedure of choice for three age categories of patients with BMIs over 50 kg/m2 as reported by the participants of the survey. **A** Under 18 years old, **B** 18 to 65 years old, **and C** older than 65 years old. *LAGB* laparoscopic adjustable gastric banding, *LSG* laparoscopic sleeve gastrectomy, *LRYGB-S* standard Roux-en-Y gastric bypass, *LRYGB-LAL* Roux-en-Y long alimentary limb > 100 cm gastric bypass, *LRYGB-LBL* Roux-en-Y long biliary limb > 150 cm gastric bypass, *OAGB-F* one anastomosis gastric bypass with fixed limb measures, *OAGB-T* one anastomosis Gastric Bypass with tailored limb measures, *SADI-S* single anastomosis duodeno–ileal bypass with sleeve gastrectomy, *BPD-DS* biliopancreatic diversion with duodenal switch

**Fig. 2:**
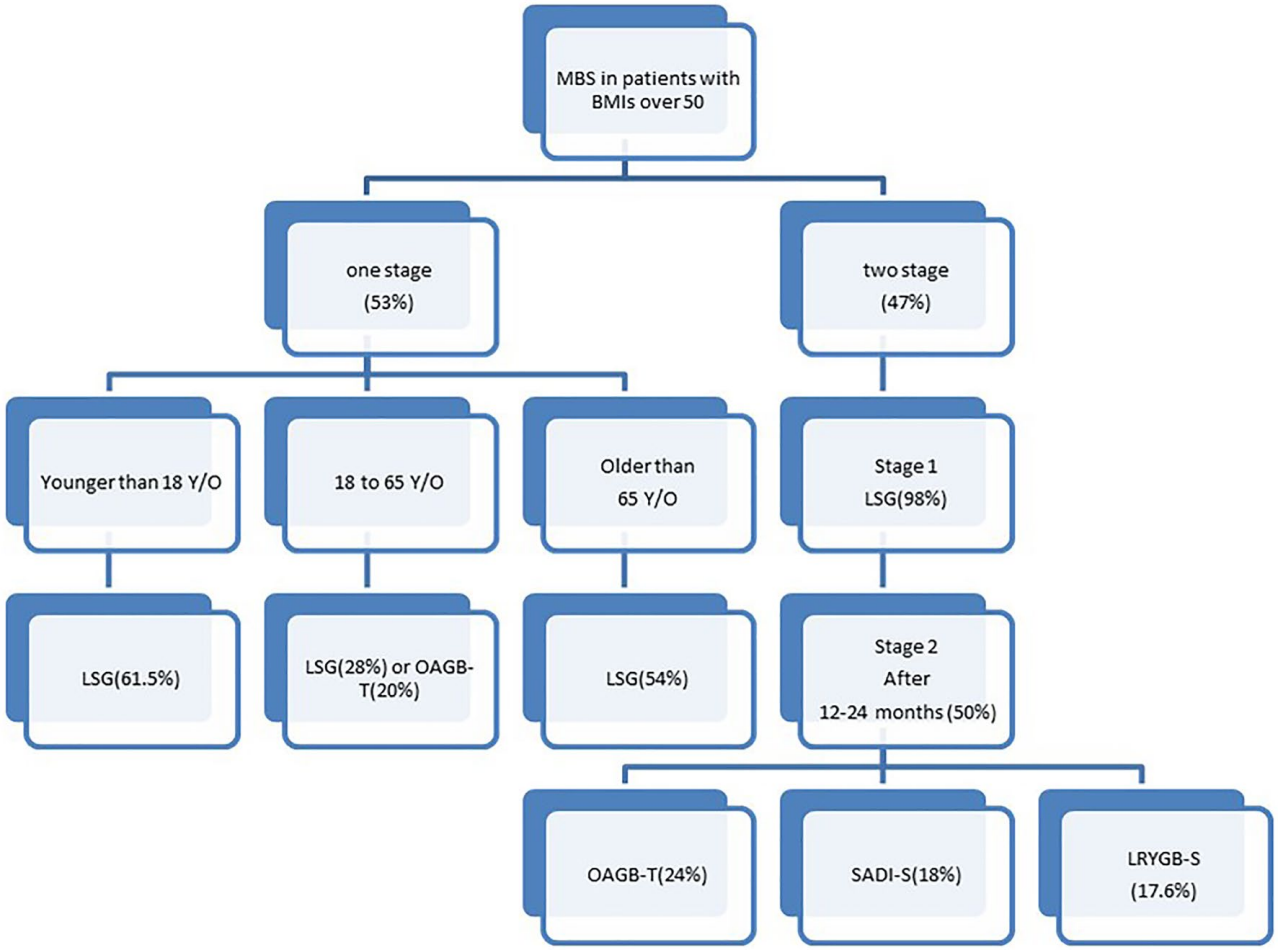
Metabolic and bariatric surgeries (MBS) algorithm in patients with BMIs over 50 based on respondent's answers. The percentage of the most responses of each items was mentioned. *LSG* laparoscopic sleeve gastrectomy, *LRYGB-S* standard Roux-en-Y Gastric Bypass, *OAGB-T* one anastomosis gastric bypass with tailored limb measures, *SADI-S* single anastomosis duodeno–ileal bypass with sleeve gastrectomy, *BPD-DS* biliopancreatic diversion with duodenal switch

SG was considered the best choice for patients younger than 18 (62.1%) or older than 65 years old (54.7%). SG and OAGB were the most chosen procedures for individuals between 18 and 65 years (28% vs 20.6%, respectively) (Figs. 1, 2). There was no consensus regarding limb lengths and total small bowel measurement. Almost half of respondents (45.9%) recommended a 2-stage approach. SG should be the first step for 98% of participants, while LAGB only for 1.8%. As second stage, the OAGB with tailored limb, the SADI-S, the standard RYGB, and the RYGB with a long biliary limb over 150 cm were chosen by 24.5%, 18.4%, 16.5%, and 14.2%, respectively. Other options for second stage procedure were the OAGB with fixed limb (9.8%), the RYGB with long alimentary limb over 100 cm (5.5%), and the BPD/DS (3.8%). A small percentage (6.9%) of participants perform other surgical procedures.

Most participants (62.5%) stated that they had not performed robotic bariatric surgery on patients with BMIs over 50 kg/m^2^. However, 25.1% of participants who were experienced in robotic bariatric surgery believed that robotic surgery did not decrease postoperative morbidity in these patients. Only 3.3% of respondents considered single-incision laparoscopic surgery (SILS) to be safe for these patients, while 73% advised against it.

Thirty percent of participants recommended that revisional surgery or conversion for poor weight loss in patients should always be done, but most of them (68.6%) advised to perform an extensive preoperative evaluation.

Half of the participating surgeons (50.4%) recommended a time interval between the first and second bariatric procedures of 12 to 24 months. However, other surgeons would perform the second surgical procedure after less than 6 months (6.3%), 6–12 months (20.8%) and more than 24 months (22.3%).

The majority of participants (76.5%) believe that if there is resolution of comorbidities and adequate weight loss after the first procedure, performing the second step of the two-step procedure is not recommended.

When a micro/macro-nodular liver cirrhosis is found, 60.7% of surgeons perform liver biopsies, while 8.3% perform biopsies routinely and 14.5% only in case of fatty liver; 16.3% declared that they never perform biopsies during MBS.

### Postoperative management (Online Appendix 4)

The majority of participants, 567 of the 789 surgeons (71.8%), did not recommend any postoperative imaging (upper gastrointestinal series, CT scan, etc.). 122 surgeons (15.5%) do believe that imaging should be performed routinely before hospital discharge and another 100 (12.6%) of participants believe that imaging should be done shortly after surgery.

Almost eighty percent (78.7%) of participants recommended postoperative intensive care unit admission for selected cases. Serum levels of creatine kinase to exclude rhabdomyolysis were routinely assessed by 43.6%, while 41.3% of participants measured it only when the intervention lasted longer than 2 h.

Postoperative thromboprophylaxis was recommended for 2 weeks by 37.8% of participants and for 4 weeks by 37.7%; 27.8% of surgeons prescribe postoperative anticoagulation for only one week. Postoperative vitamin supplementation was considered necessary by 95.6% of respondents.

According to 65.1%, there was no need to shorten the interval for postoperative follow-up appointments.

### Definition of weight loss failure and weight loss outcome

The most frequent response (68.6%) for defining weight loss failure after MBS was weight loss lower than 50% of excess weight. Other responses included BMI over 40 kg/m^2^ (14.2%) and BMI over 35 kg/m^2^ (11.6%). Most surgeons (44.2%), believed that %EWL was a more accurate index for reporting weight loss outcomes compared to TWL (19.8%) or EBMIL (19.8%). Almost thirty-seven percent of respondents felt that the definition of ideal body weight for patients with BMIs over 50 kg/m^2^ was a BMI of 30 kg/m^2^. Data are summarized in online Table 6.

### Interval to other types of surgery

Furthermore, the top three-time intervals surgeons considered appropriate for weight stabilization so that patients with BMIs over 50 kg/m^2^ could undergo other types of surgery was: 12 months, 18 months and 24 months for 39.8%, 32.3%, and 21.3% of respondents, respectively.

## Discussion

Patients with BMI over 50 kg/m^2^ are unanimously considered high-risk candidates for MBS. Indeed, surgery on these patients should be preferably performed by expert bariatric surgeons with the support of experienced bariatric anesthesiologists [16]. However, in this survey, most surgeons responded that they did not use a different surgical risk score for these patients.

A recent study revealed that BMI over 50 kg/m^2^ was independently associated with female sex and left ventricle hypertrophy but not with hypertension, diabetes, or a higher rate of surgical complications [17]. This may explain why 66.1% of surgeons in this survey acknowledged that BMI over 50 kg/m^2^ is an indication for thorough preoperative cardiac evaluation.

Interestingly, most respondents believed that it is safe and effective to perform bariatric surgery in both elderly and adolescents with BMI over 50 kg/m^2^ [18–20].

Several studies have showed that after MBS, patients with preoperative eating disorders lost significantly less weight than patients without. Preoperative assessment and interventions targeting psychosocial dysfunction could decrease eating disorder symptoms [21, 22]. This was supported in the present survey as 73.7% of respondents routinely perform preoperative eating disorder evaluation and psychological assessment.

Despite that a clear majority of respondents support preoperative weight loss for patients with BMI over 50 kg/m^2^, there is no consensus on how to reach this goal. Very few surgeons advised to insert an IGB and VLCDs were not routinely recommended due to bad compliance from bariatric subjects. However, a systematic review showed that a low-calorie diet (LCD, 800–1200 kcal), instead of a very-low-calorie diet (VLCDs, 450–800 kcal per day), for 2 to 4 weeks should be preferred [23]. Indeed, individuals on VLCDs may experience symptoms like fatigue, headache and nausea compromising the compliance [23], but another systematic review recommended that VLCD should be used for 4–8 weeks prior to surgery [24].

Consistent with previous publications in the literature, the respondents’ opinion of the benefits of preoperative weight loss on outcomes was inconsistent. There is not enough high quality evidence showing benefits of preoperative weight loss [25]. Further validation may need to be carried out. The results of this survey were also controversial as 39% of surgeons believe that the amount of weight loss was not important for proceeding with surgery.

Moreover, it is universally accepted that bariatric surgery in patients with BMIs over 50 kg/m^2^ results in less weight loss and more complications when compared to patients with BMIs less than 50 kg/m^2^. Therefore, for patients with obesity and BMIs > 50 kg/m^2^ it is very important to offer these patients procedures that are safe and effective in the long term [26–28]. In this view, it is interesting that most surgeons in this survey preferred to perform SG on adolescents, as also suggested by current literature [29].

Procedure selection in patients between the ages of 18 to 65 years maybe more flexible as there are many published studies demonstrating safety and efficacy in this range of ages. Most surgeons participating in this study prefer SG and OAGB. Some studies have reported that SG is a safe and effective one-stage procedure for patients with BMIs over 50 kg/m^2^ [30, 31]. However, other studies have reported that SG results in insufficient weight loss in these patients and should only be used as first-stage operation of a 2-stage gastric bypass or BPD/DS [32–36]. Thereaux et al. concluded that SG can be an independent factor for weight loss failure in patients with BMIs over 50 kg/m^2^ [37].

The OAGB with a tailored limb length of about 200 cm has shown to achieve good weight loss for patients with BMIs over 50 kg/m^2^ [38] and has shown superiority when compared to the SG [39–41, 42]. A systematic review by Parmar et al. reported mean %EWL of 90.75%, three years after OAGB patients with BMIs over 50 kg/m^2^ [43]. Additionally, Miller et al. reported %EWL of 72.2% five years after a banded OAGB in these patients [44].

Some authors have recommended RYGB as a safe and effective procedure in patients with BMI over 50 kg/m^2^, since it achieves better weight loss than SG [31, 45–50]. Obeid et al. reported a 52.9%EWL at 10 years after RYGB [51]. Some studies have recommended creating the RYGB with longer biliopancreatic limbs (BPL) in RYGB for patients with BMIs over 50 kg/m^2^ to achieve better weight loss and lower rates of weight regain [52, 53]. Only 11.1% of surgeons create their RYGB with long BPL over 150 cm for these patients. There is no consensus concerning whether a longer alimentary limb (AL) results in better weight loss in patients with BMIs over 50 kg/m^2^. A systematic review by Orci et al. found that RYGB with a long AL over 100 cm had better efficacy for patients with severe obesity [54], but Sarhan et al. showed that longer ALs, had no significant effect on weight loss and weight regain was noted at 3 years follow-up [55].

The BPD/DS maybe the most effective bariatric procedure for patients with BMIs over 50 kg/m^2^ as primary procedure [56, 57] or as conversion after primary SG [58]. However, it may have higher rates of malnutrition, diarrhea and fecal incontinence [59]. In recent studies, the SADI-S procedure has also been shown to achieve good results in patients with BMIs over 50 kg/m^2^ as a one [56, 60] or two-stage procedure [61–63]. As expected, the LAGB is the least effective procedure for patients with BMIs over 50 kg/m^2^ and should not be recommended as primary procedure [46, 64, 65].

Advanced age and high BMIs are risk factors for weight loss failure and weight regain after bariatric surgery [65, 66]. Additionally, elderly patients may be high risk for greater postoperative morbidity and mortality [28, 67]. SG is the most commonly performed bariatric/metabolic procedure by participating surgeons (54.7%) for elderly patients with BMIs over 50 kg/m^2^, as also supported by current literature [68]. Other standard procedures such as RYGB, OAGB, BPD/DS and revisional procedures have been shown to be effective but may have greater morbidity compared to younger patients [38, 67, 69, 70].

Surprisingly, only 1 out of 3 surgeons routinely measure the length of the entire small intestine during any type of gastric bypass in patients with BMI over 50 kg/m^2^. Abellan et al. showed that small bowel length during RYGB had no effect on weight loss in this group of patients. However, when creating longer AL, measurement of small bowel length may be necessary to assure that the common limb will be greater than 50% of total bowel length to prevent nutritional deficiencies [71]. Ahuja et al. recommended total bowel length measurement during OAGB where the BPL limbs will be 250 cm or more [40]. It seems reasonable that total bowel length should be measured for any gastric bypass with long limbs.

This survey found that 54% of surgeons perform one-stage bariatric surgery in this patient population, while 45.9% prefer a two-stage approach. Some evidence suggests that a two-stage surgical approach may decrease morbidity and mortality in patients with BMIs over 50 kg/m^2^ [27, 35]. SG is recommended as the first-stage procedure due to its simplicity and shorter operative time [35]. About 98.2% of surgeons participating in this study supported this approach. For the second stage, most of participants performed OAGB with tailored limb lengths. SADI-S, standard RYGB and RYGB with a long biliary limb over 150 cm were less commonly performed. Only 3.9% of surgeons would perform BPD/DS as second stage procedure for patients BMI greater than 50 kg/m^2^. Safety and efficacy of conversional surgeries have been confirmed by several studies [26, 27, 32, 35, 58, 61–63]. Moreover, as reported in previous published studies [27, 32, 35, 61, 63], there was a wide time interval between the two stages.

Robotic surgery for severely obese patients provides ergonomic advantages for the surgeon, but without any documented clinical advantages when compared to laparoscopic approaches [15, 72]. However, vast majority of respondents did not support the use of the robot for subjects with BMI superior to 50 kg/m^2^.

Similarly, despite single-incision laparoscopic SG was reported by Pourcher et al. [12] with acceptable outcomes and low complication rates (4.8%), participants of this survey do not recommend SILS for individuals with severe obesity.

Intraoperative liver biopsy has been a matter of contextual debate among bariatric and metabolic surgeons. In this survey, 61% of participants prefer to biopsy the liver only if micro- or macro-nodular cirrhosis is observed intraoperatively rather than routine biopsy. A systematic review of 27 studies on liver biopsy during bariatric surgery (regardless of BMI) showed that 25% of patients had NASH with a large degree of heterogeneity [73]. Bedossa et al. reported that in a cohort of 798 patients with severe obesity, older age as well as visceral adiposity may play a more relevant role in NASH than high BMI [74].

In this survey, 79% surgeons believed that ICU is not mandatory for all patients with BMI more than 50 kg/m^2^ and it is only necessary for selected patients. However, two studies demonstrated higher unplanned ICU admission rate among these subjects. Both studies used a national database, the American College of Surgeons Metabolic and Bariatric Surgery Accreditation and Quality Improvement Program (MBSAQIP) Data Registry. Both studies divided patients into BMI greater than 50 kg/m^2^ and BMI greater than 60 kg/m^2^. Wilkinson et al. [75] analyzed the outcomes of over 30,000 patients with BMI over 50 kg/m^2^ and Nasser et al. [76] analyzed the outcomes of over 85,000 patients BMI over 50 kg/m^2^. Both studies confirmed that the frequency of unplanned ICU care was higher than for patients with severe obesity.

This survey found that 72.2% of surgeons favored postoperative prophylactic anticoagulation for at least 2 weeks (up to 6 weeks). This practice is supported by published data by Wilkinson et al. [75] and Nasser et al. [76], who reported higher incidences of DVT/PTE among patients with BMI superior to 50 kg/m^2^. In addition, Stier et al. demonstrated that patients with morbid obesity (at least those with BMI greater than 50 kg/m^2^) treated with routine prophylactic enoxaparin did not achieve the defined target range for aFXa [77].

More than 95% of participants in this survey believed that patients with BMI more than 50 kg/m^2^ need postoperative vitamin supplementation. In addition, 65% of surgeons supported shorter time intervals between follow-up visits for patients with high BMIs. Apparently, they believe that a higher degree of postoperative care is necessary for patients with BMIs greater than 50 kg/m^2^ as compared to patients with morbid obesity.

Predictive factors for rhabdomyolysis after bariatric surgery are: patients having a BMI superior to 40 kg/m^2^, patients classified as ASA III or IV physical status, diabetics and those whose operation lasts longer than 4 h [78]. Similarly, a systematic review by Chakravartty et al. showed that patients with higher BMIs, male patients and those who had prolonged surgery are at greater risk of rhabdomyolysis [79]. The above-mentioned studies have proposed measuring serum CK levels in high-risk patients [78, 79]. In addition, another cohort of 485 patients reported an increased risk of rhabdomyolysis when the duration of surgery was greater than 230 min and suggested the serum CK should be checked for all bariatric patients within the first 24 h postoperatively [80]. Considering the lack of sufficient evidence regarding CK measurements, it is not surprising that in this survey there was no consensus. 43.6% of surgeons did not measure CK routinely, while 41.4% checked CK only when the operative time exceeded 2 h.

The ASMBS guidelines advocate the use of %EWL as an item for weight loss outcomes reporting [81]. Usually the Reinhold's criteria [82], with definition of excess weight loss less than 50% or BMI > 35 for insufficient weight loss is used as the definition of weight loss failure. However, previous studies supported the use of “initial weight” and “ideal weight” as mandatory items for %EWL calculation. However, they are not clearly defined and can cause outcome variability [81]. Although “ideal weight” is also considered as BMI 25 kg/m^2^ by the ASMBS, another study proposed that for patients with BMI more than 50 kg/m^2^, BMI 25 could not reflect “ideal weight” because of wide range between initial weight and ideal weight [83]. In this survey, despite 68.8% agreement on %EWL less than 50% for weight loss failure, once again significant diversity was observed regarding ideal weight. More studies clarifying definitions for ideal weight, and initial weight are needed for patients with BMI greater than 50 kg/m^2^.

Some papers revealed improved outcomes of lower extremity joint replacements after bariatric surgeries [84, 85], while in contrast other reports found no significant reduction in the incidence of complications of lower extremity joint replacements after bariatric operations [86]. The interval time between the initial bariatric operation and joint replacement (knee or hip) surgery was evaluated by Schwarzkopf et al. He recommended waiting at least 6 months after bariatric surgery before proceeding with joint replacement surgeries [87]. However, respondents of this survey believed that a time interval of 12 months was sufficient.

This first survey addressing MBS on patients with BMI over 50 kg/m^2^ provides common ground regarding different aspects of this hot topic. First, MBS in this subgroup of patients should only be performed by expert bariatric surgeons and only experienced bariatric anesthesiologists should be involved. Second, no surgical risk scoring system is used for the moment for these patients. Third, MBS seems to be safe for all age groups of patients with a BMI over 50 kg/m^2^, including adolescents (under 18 years old) and elderly (over 65 years old) patients. Fourth, patients should be routinely assessed for preoperative eating and psychological disorders. Fifth, preoperative and postoperative CPAP should be used in selected cases with sleep apnea. Sixth, SG is considered by far the best choice for patients younger than 18 or older than 65 years old with BMI over 50 kg/m^2^. Seventh, revisional surgery or conversion for poor weight loss in these patients should only be performed after extensive preoperative evaluation. Eighth, in a two-stage MBS approach in this subgroup of patients, if resolution of comorbidities and adequate weight loss after the first procedure is achieved, then performing the second step of the two-step procedure is not recommended. Ninth, when a micro/macro-nodular liver cirrhosis is found, performing a liver biopsy is recommended. Tenth, postoperative imaging is not mandatory. Eleventh, postoperative intensive care unit admission for selected cases is recommended. Finally,

weight loss failure definition after MBS on patients with BMI over 50 kg/m^2^ is weight loss lower than 50% of excess weight.

### Limitations and Strength of Study

IFSO currently counts more than 6500 members, and while the vast majority of them are bariatric surgeons only 11.8% (769) participated in this survey. Subsequently, a minority of members have responded to our questionnaire. However, to date, this is the largest survey addressing perioperative management on subjects with BMI over 50 kg/m^2^.

## Conclusion

This survey is the first survey addressing the issues concerning patients with BMI over 50 kg/m^2^ and showed significant variations in the management of bariatric surgery for this class of obesity. An international consensus on this topic should be carried out.

## Supplementary Information

Below is the link to the electronic supplementary material.

Supplementary file1 (DOCX 17 kb)

Supplementary file2 (DOCX 15 kb)

Supplementary file3 (DOCX 13 kb)

Supplementary file4 (DOCX 12 kb)

Supplementary file5 (DOCX 13 kb)

Supplementary file6 (DOCX 13 kb)
