# Supplementary material for: The first survey addressing patients with BMI over 50: a survey of 789 bariatric surgeons
Source: Surg Endosc. 2022 Jan 21;36(8):6170–80. doi: 10.1007/s00464-021-08979-w (PMC9283149; doi:10.1007/s00464-021-08979-w)
Supplement: Supplementary file 1 — Supplementary file1 (DOCX 17 kb) [file 464_2021_8979_MOESM1_ESM.docx]

**Table 1** Survey questionnaire with summary of responses

| Questions | Responses |  |  |  |
| --- | --- | --- | --- | --- |
| PART 1 - PREOPERATIVE GENERAL EVALUATIONS AND MANAGEMENTS | | | | |
| Are you a bariatric surgeon? | Yes | No |  |  |
| How many metabolic and bariatric surgeries (MBS) have you done till now? | Less than 100 | 100 to 500 | 501 to 1000 | More than 1000 |
| Which country do you work in? |  |  |  |  |
| MBS on patients with BMIs over 50 should only be performed by fully trained bariatric surgeons? | Yes | No | Maybe |  |
| Only an experienced bariatric anesthetist should anesthetize patients with BMIs over 50? | Yes | No | Maybe |  |
| Do you use any surgical risk score in patients with BMIs over 50 that is different from the usual pre-anesthetic scores? | Yes | No |  |  |
| Should patients with BMIs over 50 undergo surgery exclusively in Accredited Bariatric Surgery Centers? | Yes | No |  |  |
| Postoperative imaging (upper gastrointestinal series, CT scans): | Should be routinely performed in the immediate postoperative period | Should be routinely performed before discharge | Should not be performed routinely |  |
| Should patients with BMIs over 50 undergo MBS on: | Ambulatory basis (discharge the same day after surgery) | Fast track basis (POD 1) | Discharge on POD 2 | Discharge on POD 3-5 |
| Should MBS on patients with BMIs over 50 be declared as ''urgent bariatric surgery"? | Yes | No |  |  |
| Do patients with BMIs over 50 need a different or more thorough multidisciplinary evaluation? | Yes | No |  |  |
| Is BMIs over 50 an indication for complete preoperative cardiac evaluation in absence of other indications? | Yes | No |  |  |
| Should patients with BMIs over 50 who smoke be offered MBS? | Yes, it doesn’t matter | No, I do not operate on smokers | On a case by case basis |  |
| Are there differences in perioperative management between patients with BMI 50, 60 or 70? | Yes | No | Maybe |  |
| Are there differences in perioperative management for patients with BMIs over 50 regarding waist circumference and/or comorbidities? | Yes | No | Maybe |  |
| Would you perform MBS on patients with BMIs over 50 > 65 years? | Yes | No |  |  |
| Would you perform MBS on patients with BMIs over 50 <18 years? | Yes | No |  |  |
| Should all patients with BMIs over 50 undergo preoperative eating disorder and psychological assessment? | Always | Never | In selected cases |  |
| PART 2 - PREOPERATIVE WEIGHT LOSS MANAGEMENTS | | | | |
| Is a preoperative Intragastric Balloon recommended for patients with BMIs over 50? | Always | Never | In selected cases |  |
| Is preoperative Liraglutide recommended in for patients with BMIs over 50? | Always | Never | In selected cases |  |
| Should patients with BMIs over 50 be prepared prior to surgery in an ambulatory or hospital setting (i.e. Stier-Regime)? | Ambulant | Hospital | No difference |  |
| Do you administer an extended preoperative liver shrinkage diet (VLCD) to patients with BMIs over 50 (more than 4 weeks)? | Yes | No | Only in patients with hepatomegaly |  |
| Regarding preoperative diet: | Weight loss is mandatory to proceed with surgery | Weight loss must be encouraged before proceeding with surgery | Patients should not be submitted to a preoperative diet |  |
| Regarding preoperative diet: | An excess weight loss of 10% is recommended to proceed with surgery | An excess weight loss of 10-20% is recommended to proceed with surgery | An excess weight loss of more than 20% is recommended to proceed with surgery | Amount of excess weight loss is not important |
| Do you recommend that all patients with BMIs over 50 use pre and post-operative CPAP? | Yes | No | Only in selected cases with sleep apnea |  |
| What should be the procedure of choice for adolescents with BMIs over 50 (under 18 years old)? | Data summarized in Figure 1 |  |  |  |
| What should be the procedure of choice for patients with BMIs over 50, 18-65 years old? |  |  |  |  |
| What should be the procedure of choice for patients with BMIs over 50 older than 65?* |  |  |  |  |
| Do you recommend that the entire small intestine be measured during any form of gastric bypass in patients with BMIs over 50? | Yes | No | Maybe |  |
| Is a two-stage approach recommended for patients with BMIs over 50? | Yes | No |  |  |
| Should patients with BMIs over 50 be offered revisional or conversion surgery for poor weight loss after their primary MBS? | Yes, always | No, never | Only certain patients after extensive evaluation |  |
| If recommend 2 stage approach, which first stage procedure would you recommend? | Adjustable Gastric Band | Sleeve gastrectomy |  |  |
| Which two stage procedure would you recommend for patients with BMIs over 50? |  |  |  |  |
| What should be the time lapse between the first and second procedure? | 6 months | 6-12 months | 12-24 months | > 24 months |
| Should a two stage operation be performed if the patient achieved comorbidities resolution and adequate WL after the first stage operation? | Yes | No |  |  |
| PART 3 - SURGICAL TECHNICAL DETAILS | | | | |
| Do you recommend investigational procedures in patients with BMIs over 50? | Yes | No | Maybe |  |
| Does robotic bariatric surgery decrease postoperative morbidity and/or in patients with BMIs over 50? | Yes | No | I don't have experience |  |
| Is single incision surgery (SILS) suitable and safe for patients with BMIs over 50? | Yes | No | Maybe |  |
| Do you recommend patients with BMIs over 50 be admitted to the ICU after MBS? | Always | Never | In selected cases |  |
| Is liver biopsy during MBS necessary for patients with BMIs over 50? | Always | Never | If fatty liver is found during surgery | If micro or macronodular liver cirrhosis is found during surgery |
| PART 4 - POSTOPERATIVE MANAGEMENT | | | | |
| How long would you recommend anti-coagulant usage in patients with BMIs over 50? | One week | 2 weeks | 4 weeks | 6 weeks |
| Is postoperative vitamin supplementation necessary for all patients with BMIs over 50 undergoing MBS? | Yes | No |  |  |
| Should post-operative follow-ups and para-clinical assessments be done with shorter interval in comparison to patients with BMIs below 50? | Yes | No |  |  |
| Do you recommend to measure creatine kinase (CK) to exclude rhabdomyolysis in postoperative course in patients with BMIs over 50? | Yes | No | Only in surgeries with operative time more than 2 hours |  |
| What is the definition of weight loss failure for patients with BMIs over 50 after 2 years? | BMI >35 kg/m2 | BMI >40 kg/m2 | Less than 50% EWL | Less than 60% EWL |
| Which factor may be more accurate for reporting weight loss outcomes for patients with BMIs over 50? | EWL | TWL | EBMIL |  |
| Ideal body weight for patients with BMIs over 50, should be defined based on BMI of...? | 25 Kg/m2 | 30 Kg/m2 | 35 Kg/m2 | 40 Kg/m2 |
| How long do you think it is appropriate to wait for weight stabilization in a patient with BMIs over 50 in order to perform other types of surgery? (arthroplasty, knee replacement, abdominal wall hernias, dermolipectomies, etc.) | 12 months | 18 months | 24 months | >24 months |
|  |  |  |  |  |
|  |  |  |  |  |
