# Supplementary material for: The first survey addressing patients with BMI over 50: a survey of 789 bariatric surgeons
Source: Surg Endosc. 2022 Jan 21;36(8):6170–80. doi: 10.1007/s00464-021-08979-w (PMC9283149; doi:10.1007/s00464-021-08979-w)
Supplement: Supplementary file 2 — Supplementary file2 (DOCX 15 kb) [file 464_2021_8979_MOESM2_ESM.docx]

**Table 2:** Country of Origin of Respondents in Alphabetical Order

| Country of Origin | Number of Responses | Percentage |
| --- | --- | --- |
| Argentina | 62 | 7.86% |
| Australia | 12 | 1.52% |
| Austria | 2 | 0.25% |
| Azerbaijan | 1 | 0.13% |
| Bahamas | 1 | 0.13% |
| Belgium | 12 | 1.52% |
| Bolivia (Plurinational State of) | 8 | 1.01% |
| Brazil | 40 | 5.07% |
| Bulgaria | 1 | 0.13% |
| Cabo Verde | 1 | 0.13% |
| Canada | 1 | 0.13% |
| Chile | 5 | 0.63% |
| China | 75 | 9.51% |
| Colombia | 16 | 2.03% |
| Costa Rica | 2 | 0.25% |
| Croatia | 1 | 0.13% |
| Dominican Republic | 3 | 0.38% |
| Ecuador | 7 | 0.89% |
| Egypt | 18 | 2.28% |
| Finland | 2 | 0.25% |
| France | 59 | 7.48% |
| Germany | 22 | 2.79% |
| Greece | 6 | 0.76% |
| Guatemala | 3 | 0.38% |
| Iceland | 1 | 0.13% |
| India | 15 | 1.90% |
| Iran (Islamic Republic of) | 47 | 5.96% |
| Iraq | 6 | 0.76% |
| Israel | 4 | 0.51% |
| Italy | 46 | 5.83% |
| Japan | 5 | 0.63% |
| Jordan | 3 | 0.38% |
| Kazakhstan | 1 | 0.13% |
| Korea, South | 1 | 0.13% |
| Kuwait | 3 | 0.38% |
| Lebanon | 6 | 0.76% |
| Malaysia | 4 | 0.51% |
| Mexico | 50 | 6.34% |
| Morocco | 3 | 0.38% |
| Netherlands | 7 | 0.89% |
| New Zealand | 1 | 0.13% |
| Norway | 3 | 0.38% |
| Pakistan | 2 | 0.25% |
| Peru | 12 | 1.52% |
| Philippines | 2 | 0.25% |
| Poland | 5 | 0.63% |
| Portugal | 9 | 1.14% |
| Qatar | 2 | 0.25% |
| Romania | 1 | 0.13 |
| Russian Federation | 4 | 0.51% |
| Saudi Arabia | 14 | 1.77% |
| Singapore | 2 | 0.25% |
| Slovenia | 1 | 0.13% |
| South Africa | 1 | 0.13% |
| Spain | 37 | 4.69% |
| Sudan | 1 | 0.13% |
| Sweden | 9 | 1.14% |
| Switzerland | 5 | 0.63% |
| Syrian Arab Republic | 1 | 0.13% |
| Taiwan | 3 | 0.38% |
| Thailand | 4 | 0.51% |
| Tunisia | 1 | 0.13% |
| Turkey | 20 | 2.53% |
| Ukraine | 4 | 0.51% |
| United Arab Emirates | 11 | 1.39% |
| United Kingdom | 28 | 3.55% |
| United State of America | 37 | 4.69% |
| Uzbekistan | 1 | 0.13% |
| Venezuela(Bolivarian Republic of) | 4 | 0.51% |
| Yemen | 1 | 0.13% |
| Total | 789 | 100% |
