# Supplementary material for: The first survey addressing patients with BMI over 50: a survey of 789 bariatric surgeons
Source: Surg Endosc. 2022 Jan 21;36(8):6170–80. doi: 10.1007/s00464-021-08979-w (PMC9283149; doi:10.1007/s00464-021-08979-w)
Supplement: Supplementary file 3 — Supplementary file3 (DOCX 13 kb) [file 464_2021_8979_MOESM3_ESM.docx]

**Table 3.** Preoperative management in patients with BMIs over 50 as reported by the participants of the survey

| Questions | Responses  Number of participants (percentage) | | | |
| --- | --- | --- | --- | --- |
| Do you use any surgical risk score in patients with BMIs over 50 that is different from the usual pre-anesthetic scores? | Yes  164(20.79%) | **No**  **625(79.21%)** |  |  |
| Is BMIs over 50 an indication for complete preoperative cardiac evaluation in absence of other indications? | **Yes**  **522(66.16%)** | No  267(33.84%) |  |  |
| Should patients with BMIs over 50 who smoke be offered MBS? | Yes, it doesn’t matter  160(20.28%) | No, I do not operate on smokers  253(32.07%) | **On a case by case basis**  **376(47.66%)** |  |
| Are there differences in perioperative management between patients with BMI 50, 60 or 70? | **Yes**  **362(45.88%)** | No  206(26.11%) | Maybe  221(28.01%) |  |
| Are there differences in perioperative management for patients with BMIs over 50 regarding waist circumference and/or comorbidities? | **Yes**  **356(45.12%)** | No  240(30.42%) | Maybe  193(24.46%) |  |
| Should all patients with BMIs over 50 undergo preoperative eating disorder and psychological assessment? | **Always**  **553(73.73%)** | Never  3(0.4%) | In selected cases  194(25.87%) |  |
| Do you recommend that all patients with BMIs over 50 use pre and post-operative CPAP? | Yes  102(13.60%) | No  74(9.87%) | **Only in selected cases with sleep apnea**  **574(76.53%)** |  |
|  |  |  |  |  |
