# Supplementary material for: The first survey addressing patients with BMI over 50: a survey of 789 bariatric surgeons
Source: Surg Endosc. 2022 Jan 21;36(8):6170–80. doi: 10.1007/s00464-021-08979-w (PMC9283149; doi:10.1007/s00464-021-08979-w)
Supplement: Supplementary file 4 — Supplementary file4 (DOCX 12 kb) [file 464_2021_8979_MOESM4_ESM.docx]

**Table 4.** Preoperative weight loss management in patients with BMIs over 50 as reported by the participants of the survey

| Questions | Responses  Number of participants (percentage) | | | |
| --- | --- | --- | --- | --- |
| Is a preoperative Intragastric Balloon recommended for patients with BMIs over 50? | Always  30(3.86%) | Never  261(33.59%) | **In selected cases**  **486(62.55%)** |  |
| Is preoperative Liraglutide recommended in for patients with BMIs over 50? | Always  46(5.92%) | Never  212(27.28%) | **In selected cases**  **519(66.8%)** |  |
| Do you administer an extended preoperative liver shrinkage diet (VLCD) to patients with BMIs over 50 (more than 4 weeks)? | **Yes**  **328(42.21%)** | No  268(34.49%) | Only in patients with hepatomegaly  181(23.29%) |  |
| Regarding preoperative diet: | Weight loss is mandatory to proceed with surgery  303(39%) | **Weight loss must be encouraged before proceeding with surgery**  **430(55.34%)** | Patients should not be submitted to a preoperative diet  44(5.66%) |  |
| Regarding preoperative diet: | **An excess weight loss of 10% is recommended to proceed with surgery**  **331(42.60%)** | An excess weight loss of 10-20% is recommended to proceed with surgery  114(14.67%) | An excess weight loss of more than 20% is recommended to proceed with surgery  34(4.38%) | Amount of excess weight loss is not important  298(38.35%) |
