# Supplementary material for: The first survey addressing patients with BMI over 50: a survey of 789 bariatric surgeons
Source: Surg Endosc. 2022 Jan 21;36(8):6170–80. doi: 10.1007/s00464-021-08979-w (PMC9283149; doi:10.1007/s00464-021-08979-w)
Supplement: Supplementary file 5 — Supplementary file5 (DOCX 13 kb) [file 464_2021_8979_MOESM5_ESM.docx]

**Table 5.** Postoperative management in patients with BMIs over 50 as reported by the participants of the survey

| Questions | Responses  Number of participants (percentage) | | | |
| --- | --- | --- | --- | --- |
| Postoperative imaging (upper gastrointestinal series, CT scans): | Should be routinely performed in the immediate postoperative period  100(12.67%) | Should be routinely performed before discharge  122(15.46%) | **Should not be performed routinely**  **567(71.86%)** |  |
| Should patients with BMIs over 50 undergo MBS on: | Ambulatory basis (discharge the same day after surgery)  26(3.30%) | Fast track basis  (POD 1)  244(30.93%) | **Discharge on POD 2**  **293(37.14%)** | Discharge on POD 3-5  226(28.64%) |
| Do you recommend patients with BMIs over 50 be admitted to the ICU after MBS? | Always  92(12.76%) | Never  61(8.46%) | **In selected cases**  **568(78.78%)** |  |
| How long would you recommend anti-coagulant usage in patients with BMIs over 50? | One week  201(27.88%) | **2 weeks**  **273(37.86%)** | 4 weeks  224(37.07%) | 6 weeks  23(3.19%) |
| Is postoperative vitamin supplementation necessary for all patients with BMIs over 50 undergoing MBS? | **Yes**  **680(95.64%)** | No  31(4.36%) |  |  |
| Should post-operative follow-ups and para-clinical assessments be done with shorter interval in comparison to patients with BMIs below 50? | Yes  248(34.88%) | **No**  **463(65.12%)** |  |  |
| Do you recommend to measure creatine kinase (CK) to exclude rhabdomyolysis in postoperative course in patients with BMIs over 50? | Yes  107(15.05%) | **No**  **310(43.60%)** | Only in surgeries with operative time more than 2 hours  294(41.35%) |  |
