# Supplementary material for: The first survey addressing patients with BMI over 50: a survey of 789 bariatric surgeons
Source: Surg Endosc. 2022 Jan 21;36(8):6170–80. doi: 10.1007/s00464-021-08979-w (PMC9283149; doi:10.1007/s00464-021-08979-w)
Supplement: Supplementary file 6 — Supplementary file6 (DOCX 13 kb) [file 464_2021_8979_MOESM6_ESM.docx]

**Table 6.** Weight loss outcomes definition in patients with BMIs over 50 as reported by the participants of the survey

| Questions | Responses  Number of participants (percentage) | | | |  |
| --- | --- | --- | --- | --- | --- |
| What is the definition of weight loss failure for patients with BMIs over 50 after 2 years? | BMI >35 kg/m2  85(11.61%) | BMI >40 kg/m2  106(14.48%) | **Less than 50% EWL**  **502(68.58%)** | Less than 60% EWL  39(5.33%) |  |
| Which factor may be more accurate for reporting weight loss outcomes for patients with BMIs over 50? | **EWL**  **44.2(60.38%)** | TWL  145(19.81%) | EBMIL  145(19.81%) |  |  |
| Ideal body weight for patients with BMIs over 50, should be defined based on BMI of...? | 25 Kg/m2  160(21.86%) | 30 Kg/m2  269(36.75%) | 35 Kg/m2  78(10.66%) | 40 Kg/m2  12(1.64%) | **No comment**  **21.3(29.10%)** |
| How long do you think it is appropriate to wait for weight stabilization in a patient with BMIs over 50 in order to perform other types of surgery? (arthroplasty, knee replacement, abdominal wall hernias, dermolipectomies, etc.) | **12 months**  **291(39.75%)** | 18 months  235(32.10%) | 24 months  160(21.86%) | >24 months  46(6.28%) |  |
